# Recent Advances in Antibacterial and Antiendotoxic Peptides or Proteins from Marine Resources

**DOI:** 10.3390/md16020057

**Published:** 2018-02-10

**Authors:** Zhenlong Wang, Xiumin Wang, Jianhua Wang

**Affiliations:** 1 Key Laboratory of Feed Biotechnology, Ministry of Agriculture, Beijing 100081, China; 429524082@qq.com; 2 Gene Engineering Laboratory, Feed Research Institute, Chinese Academy of Agricultural Sciences, Beijing 100081, China

**Keywords:** lipopolysaccharide, antibacterial activity, antiendotoxic activity, neutralization, marine resources

## Abstract

Infectious diseases caused by Gram-negative bacteria and sepsis induced by lipopolysaccharide (LPS) pose a major threat to humans and animals and cause millions of deaths each year. Marine organisms are a valuable resource library of bioactive products with huge medicinal potential. Among them, antibacterial and antiendotoxic peptides or proteins, which are composed of metabolically tolerable residues, are present in many marine species, including marine vertebrates, invertebrates and microorganisms. A lot of studies have reported that these marine peptides and proteins or their derivatives exhibit potent antibacterial activity and antiendotoxic activity in vitro and in vivo. However, their categories, heterologous expression in microorganisms, physicochemical factors affecting peptide or protein interactions with bacterial LPS and LPS-neutralizing mechanism are not well known. In this review, we highlight the characteristics and anti-infective activity of bifunctional peptides or proteins from marine resources as well as the challenges and strategies for further study.

## 1. Introduction

Gram-negative bacterial infectious diseases pose a major threat to humans and animals, despite the tremendous progress that has been made in medical drugs. Lipopolysaccharide (LPS), well known as an endotoxin, is a major component of the cellular wall of Gram-negative bacteria. It normally occupies up to 90% of the outer leaflet of the outer membrane and is connected by Mg^2+^ ions to form an oriented and highly ordered structure [1]. LPS is also a key signaling molecule in the pathogenesis of infection, inflammation, sepsis and multiple organ failure [2]. Septicemia is a serious disease in animal husbandry (including cattle, buffalo, pigs, fallow deer, horses, etc.) [3,4,5] that causes serious economic losses. It is also a serious human disease. Approximately 18 million sepsis cases and 8 million deaths occur in people each year [6]. Clinically, many therapeutic strategies have been applied to try to neutralize the pathogenic activity of endotoxin to prevent further deterioration of sepsis, but no satisfying therapeutic drugs exist to date [7]. Although many antibiotics have a good bactericidal effect, they cannot effectively prevent septic shock caused by LPS due to a lack of ability to neutralize LPS [8]. On the other hand, antibiotic treatment of bacterial infection often causes accelerated release of bacterial LPS [9,10]. It has been reported that a 3- to 20-fold increase in the total concentration of LPS occurs as a consequence of antibiotic action on Gram-negative bacteria [11]. Therefore, there is an urgent need for new drugs to kill bacteria and neutralize LPS at the same time. 

Antimicrobial peptides (AMPs) have received more attention due to broad-spectrum antimicrobial activity and the slow development of bacterial resistance [12]. It has been demonstrated that some AMPs from terrestrial organisms, such as LL-37, indolicidin and CAP18, possess antibacterial and antiendotoxin or anti-inflammatory properties, but few have been found in marine organisms and microorganisms [13,14,15]. In fact, the ocean is an enormous natural resource library, and marine species account for nearly half of the global diversity [16]. After long-term adaptation to its special living environment, facing infestation by marine pathogens, marine organisms are likely to metabolize many antimicrobial and antiendotoxic substances, including antimicrobial peptides and proteins that are composed of metabolically tolerable amino acids. Therefore, using these bioactive products from the ocean has a good significance for the control of pathogens and the treatment of sepsis [16,17]. 

In this review, we discussed the categories, heterologous expression, influencing factors, LPS-neutralizing mechanisms, and production limitations and the strategies of antibacterial/antiendotoxic peptides or proteins from marine resources. 

## 2. Antibacterial/Antiendotoxic Peptides or Proteins from Various Marine Organisms

### 2.1. AMPs with Different Structures

#### 2.1.1. α-Helix Peptides from Sea Snakes or Sea Breams

Many AMPs from the cathelicidin family have been found in vertebrates. Hc-CATH, the first cathelicidin identified from sea snakes, is composed of 30 amino acids with two α-helix regions in the middle and the N-terminus [18]. Hc-CATH tends to form an amphipathic α-helical conformation in bacterial membrane-mimetic environment. It has potent antimicrobial activity against G^−^ bacteria, such as *Escherichia coli*, *Vibrio*, *Klebsiella* and G^+^ bacteria, such as *Staphylococcus aureus*, *Enterococcus*, *Nocardia*, and fungi, such as *Candida* and *Arcyria* (Table 1 and Table 2). Additionally, Hc-CATH can directly bind to LPS and neutralize the toxicity, showing its anti-inflammatory activity. Due to the high stability and low cytotoxicity toward mammalian cells, Hc-CATH may be a potent candidate for the development of novel peptide antibiotics.

Chrysophsins-1, -2 and-3, which are amphipathic α-helical peptides that are rich in His residues, were isolated from the gill cells of red sea bream (*Chrysophrys major*) (Table 1 and Table 2) [19]. Chrysophsins and analogs displayed various biological activities such as antibacterial, anti-endotoxic and antitumor activities [19,20]. The C-terminal RRRH motif in chrysophsin-1 influenced the insertion of the peptide into the bacterial membranes and is crucial for pore formation and cytotoxicity [21]. A series of Chrysophsin-1 variants displayed significant antimicrobial activities against bacteria, including methicillin-resistant *S. aureus* (MRSA) strains and fungi and antiendotoxin properties by modifying its GXXXXG motif with Ala, Val or Pro [19]. These functional motifs are helpful to design novel antimicrobial and anti-endotoxin peptides. Moreover, Chrysophsin-1 and its variants displayed synergistic effects in combination with streptomycin against *E. coli* ATCC25922 and additive effects with streptomycin and chloramphenicol against *S. aureus* ATCC25923 [20]. 

Hydrostatins-TL1 and -SN1 are anti-inflammatory, active α-helical peptides, which are isolated from the venom gland of *Hydrophis cyanocinctus* in the South China Sea and can reduce inflammation in mouse models of acute lung injury and colitis [22,23,24]. 

#### 2.1.2. β-Sheet Peptides from Marine Lugworms

Arenicins-1, 2, 3 are novel peptides isolated from marine polychaeta lugworm *Arenicola marina* [25]. They are composed of 21 amino acids with one or two pairs of disulfide bonds (Cys3–Cys20 and Cys7–Cys16). Arenicins showed significant antibacterial activity against Gram-positive and Gram-negative bacterial strains (including *S. aureus*, *Enterococcus faecium*, *Staphylococcus epidermidis*, *E. coli* and *Pseudomonas aeruginosa*) as well as fungi such as *Candida albicans* (Table 1 and Table 2) [26,27,28,29,30,31]. Moreover, there is the synergistic combination between arenicin-1 and several antibiotics, such as erythromycin, chloramphenicol and ampicillin, against *E. coli*, *S. aureus*, *S. epidermidis*, and *P. aeruginosa* [27]. The arenicin-1 derivatives also showed more potent antibacterial activity against Gram-negative bacteria, such as *E. coli*, *P. aeruginosa*, and *Klebsiella pneumonia,* and Gram-positive bacteria, such as *S. aureus,* including MRSA, with the minimum inhibitory concentrations (MICs) of 0.8~50 μM and 0.06~50 μM, respectively [32,33]. Ar-1[V8R], one of the arenicin-1 variants, exhibited similar antibacterial activities, but significantly reduced cytotoxicity against mammalian cells, in contrast to arenicin-1 [34]. 

A series of arenicin-3 analogs, such as NZ17074, N2 and N6, were also designed and synthesized to improve antibacterial activity [35,36,37]. NZ17074, N2 and N6 displayed more potent antibacterial activity against *Escherichia*, *Salmonella*, *Pseudomonas*, *Staphylococcus* and *Listeria*, except *Bacillus* and *Candida*, than their parents and had a high capacity to bind to LPS by molecular docking and BODIPY’-TR-cadaverine (BC) displacement assays. These peptides could bind to LPS by forming hydrogen bonds between positively charged amino acids (such as His and Arg) and the side fatty acid chain of LPS. In addition, in vivo experiments showed that N2 and N6 significantly enhanced the survival rate of peritonitis- and endotoxemia-induced mice, indicating the antibacterial and detoxifying activity [34]. The results suggest that arenicins and their low cytotoxicity derivatives have potential as therapeutic agents and adjuvants for the treatment of bacterial infections. 

Furthermore, the antimicrobial activity of chimeric peptides of N6 and Tat11 (CNC) and the C-terminal aminated N6 (N6NH2) against *Salmonella typhimurium* in RAW264.7 cells was increased by 67.2–76.2% and 96.3–97.6%, respectively, when compared with N6 (data not published in our work). The efficacy of CNC and N6NH2 against *S. typhimurium* in mice was much higher than that of N6, suggesting that they may be excellent candidates for novel antimicrobial agents to treat infectious diseases caused by intracellular pathogens.

#### 2.1.3. Cyclic Peptides from Marine Bacteria

Novel cyclic peptides-ogipeptins, including A, B, C, and D, were isolated from the secondary metabolites of marine bacteria *Pseudoalteromonas* sp. SANK 71903 (Table 1) [38,39]. These peptides are acylated cyclic peptides with basic and hydrophobic motifs. Moreover, four ogipeptins showed strong antibacterial activity against *E. coli* with MICs of 0.25–1 μg/mL and slightly weaker activity against *S. aureus* with MICs of 8–128 μg/mL (Table 2). Meanwhile, flow cytometry analysis showed that ogipeptin could block LPS to bind to cell surface receptor CD14 in vitro with IC_50_ values of 4.8, 6.0, 4.1 and 5.6 nM, respectively. It was also found that ogipeptins were able to inhibit the release of TNF-α caused by LPS [38,39].

### 2.2. Anti-LPS Factors (ALFs) from Crustaceans

Anti-lipopolysaccharide factors (ALFs), the potential AMPs that bind and neutralize LPS, are common effectors of innate immunity in crustaceans [40]. The first ALF was isolated from amoebocytes of the horseshoe crab *Limulus polyphemus*, named LALF, which has antibacterial activity against Gram-negative bacteria and can neutralize LPS [40,41]. The first ALF of shrimp (named as ALFFc) was reported in *Fenneropenaeus chinensis* [42]. Since then, other ALFs, such as ALFP*m*3, SpALF5, and FcALF5, against different bacteria and white spot syndrome virus (WSSV) have been found to be widely distributed in different crustacean species, including mud or the green mud crabs *Scylla paramamosain*, the black tiger shrimp *Penaeus monodon*, and the Chinese shrimp *Fenneropenaeus chinensis* (Table 2) [43,44,45]. RALFPm3 could bind to lipid A of LPS [46]. Recently, a novel ALF named SpALF6, which was isolated from the mud crab *Scylla paramamosain*, and its variant SpALF6-V (H46R and A110P) possessed strong binding activity to LPS and exhibited broad antimicrobial activity against Gram-negative bacteria (such as *Vibrio* and *E. coli*), Gram-positive bacteria (such as *S. aureus*) and fungi (such as *C. albicans*) [47].

### 2.3. Other Peptides or Proteins from Sea Fish

Pardaxins (Pa1, Pa2, Pa3, and Pa4) are pore-forming membrane-active AMPs from mucous glands of sole fish. Pardaxin displayed relatively potent antibacterial activity against Gram-positive bacteria, such as *S. aureus* (1~2 μM), *S. epidermidis* (2 μM), *Bacillus megaterium* (3 μM), *Micrococcus luteus* (10 μM) and *B. subtilis* (1~5 μM), but weak activity against Gram-negative bacteria, such as *E. coli* (1~13 μM), *Acinetobacter calcoaceticus* (3 μM), *P. aeruginosa* (2~25 μM) and *S. typhimurium* (2~40 μM) (Table 2) [48,49,50]. It has been demonstrated that Pa4 can bind to LPS and adopt a unique helix-turn-helix conformation, resembling a “horseshoe” in the LPS-Pa4 complex [49]. 

Piscidins (-1, -2, and -3) are linear peptides that are isolated from hybrid striped bass. These peptides and their analogs have significant activity against Gram-negative (MICs of 0.8~25 μM) and Gram-positive (MICs of 1~16 μM) bacteria, fungi (MICs of 1.56~12.5 μM), parasites, and cancer cells (Table 2) [50,52,53,54,55,56,57]. Additionally, they also displayed potent antiendotoxin activities in vivo in mice [56]. 

The phosvitin (Pv) protein from zebrafish had potent antibacterial activity against *E. coli*, *S. aureus* and *Aeromonas hydrophila* and could bind to LPS, lipoteichoic acid or peptidoglycan of bacteria (Table 2) [58]. Pv significantly reduced LPS-induced tumor-necrosis factor (TNF)-α production in RAW264.7 cells, suggesting that Pv has LPS-neutralizing capacity in vitro. Moreover, the TNF-α level in mice was considerably decreased, and the survival of the endotoxemia mice was promoted by Pv [58]. The phosvitin-derived peptide-Pt5, which consists of the C-terminal 55 residues of zebrafish phosvitin, has antibacterial activity and immunomodulatory function. Pt5 also protected zebrafish from *A. hydrophila* infection [59]. Pt5e, one of the Pt5 mutants, showed a strong antibacterial activity against *E. coli* and *S. aureus* with MICs of 1.2–1.8 μM. Pt5e significantly inhibited the release of LPS-induced TNF-α and interleukin (IL)-1β from RAW264.7 cells and from mice, respectively [60]. Additionally, Pt5e remarkably promoted the survival of the endotoxemia mice. It also proves that Pv and its derivatives are non-cytotoxic and non-hemolytic, making them better candidates than polymyxin B and LL-37 for development as novel sepsis therapeutic agents and antibacterials. 

Zinc finger ZRANB2 protein from zebrafish can function as a pattern recognition receptor, which interacts with LPS and then recognizes Gram-negative bacteria. ZRANB2 and its truncations (Z_1/37_, Z_11/37_) exhibited bioactivity against, and bound to LPS of *E. coli*, *Vibrio anguillarum*, and *A. hydrophila* in vitro with an IC_50_ (8.5–9.7 μg/mL) (Table 2) [61]. They are also involved in the antimicrobial activity of developing embryos against *A. hydrophila* in vivo. Truncation of Z_38/198_ and the deletion of the N-terminal 37 residues of ZRANB2 resulted in losses of LPS binding activity via lipid A and antibacterial activity against Gram-negative bacteria in vitro and in vivo, indicating that the N-terminal 37 residues play a key role in the activity because of the loss of the Zn^2+^-binding site and the formation of incorrect molecular structures [61]. This provides a new viewpoint for the study of the immunological functions of zinc finger proteins. 

Ls-Stylicin1, which is characterized by a Pro-rich N-terminal region and a Cys-rich C-terminal region, was isolated from the penaeid shrimp *Litopenaeus stylirostris*. The recombinant Ls-Stylicin1 exhibited strong antifungal activity against *Fusarium oxysporum*. Additionally, it displayed potent LPS-binding activity, but lower antimicrobial activity, against *Vibrio* sp. with MICs of 40~80 μM (Table 2) [62]. 

## 3. Heterologous Expression of Antibacterial and Antiendotoxic Marine Peptides or Proteins

Arenicin-2 was fused with different partner proteins (including ketosteroid isomerase (KSI), cellulose-binding domain (CBD) and thioredoxin A) and overexpressed in *E. coli*. The yield was up to 5 mg/L (Table 3) [63]. Both recombinant and chemically synthetic arenicin-2 could inhibit Gram-positive bacteria (such as *Listeria monocytogenes*, *M. luteus*, *B. megaterium*, and *S. aureus*), Gram-negative bacteria (*E. coli* and *Agrobacterium tumefaciens*), and fungi, including *C. albicans* and spore germination of *Fusarium solani* [63]. A series of arenicin-1 variants were designed and recombinantly expressed in *E. coli*, and the yield of 1–4 mg per liter of culture was obtained. These variants exhibited low hemolysis and potent antibacterial activity against *S. aureus* (MICs of 3.13–50 μM), *E. coli* (MICs of 0.8–50 μM) and *P. aeruginosa* (MICs of 3.13–50 μM), respectively [29]. Arenicin-1 shortened analogs, ALP1 and ALP2 (17-residue), were obtained by recombinant expression in *E. coli*, with the yield of 7.5–9 mg per liter of the fermented supernatant. Both ALP1 and ALP2 improved the antibacterial activity and cell selectivity in contrast to arenicin-1 [33]. Piscidin 1 and piscidin 3 were successfully expressed in *E. coli*, with a yield of 1 mg per liter culture and with a purity of over 90% (Table 3) [64]. The recombinant Pv could significantly inhibit the growth of *E. coli*, *A. hydrophila* and *S. aureus*, with half-inhibitory concentrations (IC_50_) of 3.1, 3 and 3 μM, respectively [65]. The recombinant zebrafish phosvitin-derived peptide-Pt5 could enhance the survival of zebrafish infected with *A. hydrophila* [59]. Pv5 derivative-Pt5e was also successfully expressed in *E. coli* and displayed dual antibacterial and LPS neutralizing function in vitro and in vivo [60]. The expressed SpALF6 in *E. coli* had potent antibacterial and antifungal activity and also recognized LPS [47].

The recombinant zebrafish ZRANB2 in *E. coli* BL21 had bacterial activity against *A. hydrophila* with an IC_50_ value of 9.7 μg/mL, but the recombinant Z_38_/_198_ did not retain antibacterial activity against *E. coli*, *V. anguillarum* and *A. hydrophila* in vitro and affinity to LPS (Table 3) [61]. Moreover, recombinant ZRANB2 damaged the cell membrane integrity of *E. coli*, *V. anguillarum* and *A. hydrophila*. The yield of recombinant ALFm3 in *Pichia pastoris* is up to 118.4 mg/L and displays a broad spectrum of activity against Gram-negative bacteria (the MICs of 0.095~50 μM), Gram-positive bacteria (0.19~100 μM), filamentous fungi, such as *F. oxysporum*, *Botrytis cinerea* and *Penicillium crustosum* (1.56~25 μM), and WSSV [43,66]. The arenicin-3 varient-NZ17074 was also successfully expressed in *P. pastoris* by fusing with SUMO3, and the expressed peptide had significant antibacterial activity against *E. coli*, *S. enteritidis* and *P. aeruginosa* with MICs of 2~4, 2 and 8~16 μg/mL, respectively [67].

Low yields of these marine peptides or proteins expressed in *E. coli* and *P. pastoris* may be attributed to the antibacterial nature of peptides, which makes them potentially fatal to the hosts; additionally, their small molecular size or high cationic properties make them highly susceptible to proteolytic degradation during expression [68]. 

## 4. Factors Influencing the Interaction between LPS and Antibacterial/Antiendotoxic Marine Peptides or Proteins

### 4.1. Hydrophobicity and Charge

AMPs are usually a class of cationic amphiphilic peptides. The core oligosaccharide and phosphate group of LPS, a major component of the cell outer membrane of Gram-negative bacteria, confers a negative charge, showing its strong affinity for cationic AMPs [69,70]. How do AMPs bind to LPS and what influences their interaction? 

A large amount of LPS is released from Gram-negative bacteria and forms micelles. Positively charged AMP and negatively charged LPS will move close to each other through electrostatic interactions [71]. AMPs may insert deeply into the interior of LPS micelles by hydrophobic interactions and finally can interact with the acyl chains of lipid A through specific amino acid side chains [72]. Hydrophobic amino acids are essential for the interaction between peptides and LPS [73,74]. CLP-19, derived from Limulus ALF, and its analogs (CRP-1 and CRP-2) were chemically synthesized to assess the effects of the hydrophobic residues of peptides on the LPS-binding ability [75]. The result showed that there is a strong positive correlation between hydrophobicity and LPS binding ability, which agrees with previous studies [73,74]. 

It has been demonstrated that the positive charge of AMPs is also an important factor, which confers LPS-binding activity to peptides or proteins [70]. Scott et al. demonstrated that an increase in the positive charge in a cecropin-melittin hybrid (CEMA) significantly improved the affinity of CEMA and LPS [76]. The extra two Lys residues in the N-terminal of Sushi 3 increased the LPS-peptide binding ability [77]. A series of peptides were designed by Rosenfeld Y et al. to determine the common factors contributing to the affinity of AMPs and LPS, and the results showed that, except for an increase in the hydrophobic residues in peptides enhancing its LPS binding ability, more positively charged residues in peptides led to a stronger affinity to LPS [78]. Srivastava et al. reported that Glu substitution in Temporin L for Lys promoted the ability to bind to LPS [79]. Thus, the charge balance of the peptides is a vital parameter in the design of improved LPS-neutralizing peptides [77]. 

Additionally, the distance between the positively charged residues is very important for the binding of LPS. It was found that a typical distance range of 12~15 Å between the charged amino groups of Lys in Pa4 from sole fish and in MSI-594 (a magainin derivative) agree with the inter-phosphate distance of the phosphate groups of the lipid A domain of LPS, which is geometrically compatible to those of the AMPs and LPS conformation by NMR analysis [49,80]. Phe replacement with Ala in MSI-594 led to a loose peptide structure, which markedly reduced the affinity of LPS (4-fold) [81].

### 4.2. Basic Amino Acid Content

With the exception of hydrophobic amino acids, LPS binding to peptides or proteins also requires basic amino acids and their precise structural arrangements [73]. Kushibiki et al. used molecular docking to study the interaction between tachyplesin I and LPS and found that the amino group of Lys1 and the guanidyl group of Arg17 in tachyplesin I were in close proximity to the phosphate groups of LPS, indicating that close packing exists between the basic residues in peptides and the phosphate groups or saccharides of LPS [82]. Similarly, the indole ring of Trp2 and the aromatic ring of Phe4 of the peptide were also close to the acyl chains of lipid A, indicating that close packing also exists between the aromatic residues of tachyplesin I and the hydrophobic region of LPS [82]. 

NZ17074 and its derived peptides, N2 and N6, could bind to LPS molecules [36]. The results showed that hydrogen bonds were formed between the positively charged side chains of basic residues (His9, Arg14, and Arg19) of NZ17074 and the fatty chains MYR-1014, GLC-1007 and KDO-1003 of LPS. Salt bridges were formed between Arg10, Arg10, Arg14 and Asn21 of N2 and the fat chains FTT-1010, GMH-1005, MYR-1014 and PA1-1000 of lipid A. Arg10, Arg19 and Asn21 of N6 interacted with the aliphatic chain FTT-1010, GLC-1006 and PA1-1000 of LPS by forming hydrogen bonds or salt bridges [36]. Monisha G. Scott et al. found that, compared to CP29, CP208 interacted with LPS poorly and had little antibacterial activity due to a deletion of a Try residue at the N-terminus of CP29 [14]. 

These results indicate that basic amino acids may be very important for AMPs to neutralize LPS, which may be due to the positively charged basic amino acids that easily form hydrogen bonds with phosphate groups on the lipid head of LPS or with the lipid glycerol groups of LPS [83,84].

### 4.3. Secondary Structure

In the study of the Sushi 1 and Sushi 3 peptides, Ding et al. (2008) found that the two peptides exhibited a random structure in the aqueous phase. When interacting with LPS, Sushi 1 formed an α-helix structure, and Sushi 3 tended to form a mixture of secondary structures containing α-helix and β-sheet via an intermolecular disulfide bond, which may be contributed to its high affinity for LPS [85]. Similarly, Mozsolits et al. showed that the high α-helicity of the peptides was related to the high binding affinity with LPS. The results demonstrated that the α-helix formation of peptides plays a key role in the process of the peptides binding to LPS due to the amphipathic helical structures of the peptides [86]. Moreover, it has been reported that Pa4, which was isolated from the pacific peacock sole fish, adopts the characteristic “α-helix–loop–α-helix” structure in LPS micelles, which is essential for its interaction with LPS [49].

### 4.4. Disulfide Bond

Apart from the contribution to the structure-activity, disulfide bonds in peptides are very important for LPS binding [87]. The disulfide bonds in Sushi 1 and Sushi 3 are involved in the disruption of LPS micelles, and their reduction led to a significant decrease in the LPS-peptide binding ability by 100- and 10,000-fold, respectively, indicating that the disulfide bond play a vital role in LPS-peptide interaction [87]. A series of 12-residue β-boomerang lipopeptides, which partially contain disulfide bonds, were designed by Mohanram et al. (2014). The MIC assays showed that the incorporation of inter-molecular disulfide bond conferred strong bactericidal effects on Gram-negative bacteria. The lipopeptides analogs (YI13C, C4YI13C and C8YI13C) with disulfide bridged Cys showed 80% neutralization in different concentrations of LPS. The high-affinity interaction of peptides with negatively charged LPS are very important for the permeabilization of the cell membrane of Gram-negative bacteria and the neutralization of endotoxin [88]. 

## 5. Mechanism of Marine Peptides or Proteins in Neutralizing with LPS

### 5.1. Biophysical and Chemical Interaction with LPS

Some cationic peptides, such as SMAP-29, CAP-18 and LL-37, which contain both N- and C-terminal LPS-binding sites for the same peptide molecule, can bind to LPS via the first electrostatic interaction and via the displacement of the Mg^2+^ ions, which causes increased mobility and disordered packing of the LPS molecules and their acyl chains [89]. It has also been demonstrated that the K_5_L_7_ peptide displayed higher LPS binding capacity than its diastereomer 4D-K_5_L_7_ [1]. K_5_L_7_ bound first to LPS predominantly by electrostatic interactions and self-association, but not traverse into the hydrophobic lipid core of LPS, which confers a relatively weak LPS-permeating ability than 4D-K_5_L_7_. Comparably, after electrostatic interactions with LPS, 4D-K_5_L_7_ stays as a monomer, accumulates on the surface of the lipid core, reaches a threshold concentration and is followed by the micellization of the LPS vesicles, which indicates a more potent membrane-permeating ability than K_5_L_7_ [1]. This result is consistent with the previous study that polymyxin B could interact with LPS, including a rapid initial association and then a slower insertion into LPS [90]. The hydrophobic residues of Tachyplesin I (TP I), which was isolated from the horseshoe crab, interacted with the acyl chains of LPS. The 1D ^1^H NMR and Trp fluorescence analysis showed that the Trp2 residue of TP I is involved in binding to LPS. The docking model showed that cationic residues in the N- and C-terminal of TP I interacted with phosphate groups or saccharides of LPS and aromatic residues, such as Trp2 and Phe4 in the N-terminal of TP I, interacted with the acyl chains of LPS [82]. 

Moreover, multiple Arg or Lys residues in the peptides or proteins, such as ALFs (ALFH1, ALFH2), bactericidal/permeability increasing factor (B/PI), lactoferrin and lysozyme, may form a structural motif, which most likely binds anionic groups of lipid A or inner core polysaccharide regions of LPS [89,91]. Both Lys and Arg residues in Sushi 3 interacted with the diphosphoryl head groups of LPS, and its hydrophobic residues contributed to interactions with the acyl chains of LPS [77]. The Sushi 1 peptide could bind parallel to the acyl chains of the lipid layer [92]. The Trp residue of Sushi 1 was located in the hydrophobic acyl chains of lipid A in LPS, which resulted in a decrease in LPS interaction with other binding sensors, such as LBP in the host [92]. 

Additionally, the NMR structure of Pa4 in LPS micelles showed that the Lys residues at the positions 8 and 16 could bind to the lipid A moiety of LPS via a salt bridge or hydrogen bond [49]. These results indicate that lipid A plays a vital role in the interaction between peptides or proteins and LPS, and it is the pharmacophore of the LPS molecule [92].

### 5.2. Inhibition of LPS-Induced Inflammatory Response

**Figure 1:**
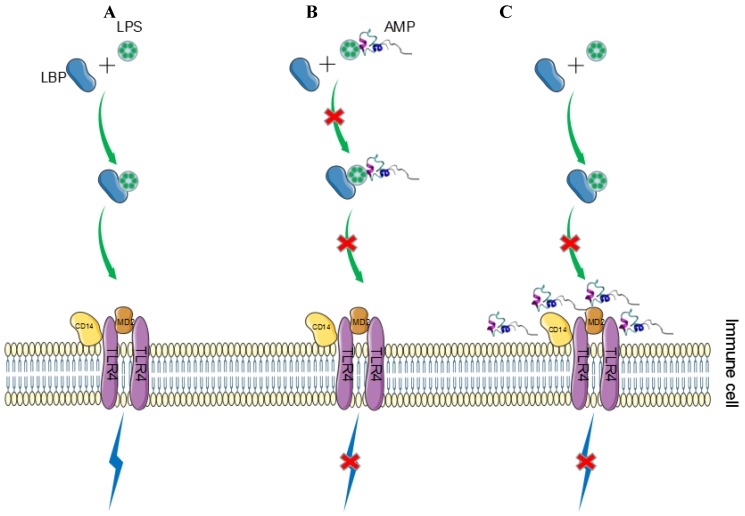
Mechanism of marine peptides or proteins in neutralizing LPS. LPS can bind to TLR4, which subsequently activates the pro-inflammatory pathways and release cytokines (**A**). Interaction of peptides or proteins with LPS may include (i) a direct effect, in which peptides or proteins directly bind to LPS, thus inhibiting the LPS-TLR4/MD2 binding and the activation of the signal pathways (**B**); and (ii) an indirect effect, in which peptides or proteins competitively bind to CD14 or TLR4, which thereby indirectly inhibit LPS-induced inflammatory response (**C**) [82,93].

How does LPS cause an inflammatory response? The released LPS in vivo can be transported to a cell membrane surface receptor-CD14 with the help of LBP (an LPS binding protein in the blood) and form the LPS-CD14 complex. LPS is then recognized by toll-like receptor 4 (TRL4) on the surface of the phagocytic cells, which in turn activates the MAPK and NF-κB pathways, which can induce the production of pro-inflammatory cytokines. The mechanism of peptides or proteins in neutralizing LPS and inhibiting the inflammatory response can be divided into (i) a direct effect: many bifunctional peptides (such as Hc-CATH) or proteins (such as SpALF6) can directly bind with LPS, which thus inhibits the binding of LPS to the TLR4/MD2 complex and the activation of inflammatory response pathways [18,47]; and (ii) an indirect effect: peptides (such as LL37 and defensin) or proteins competitively bind to CD14 or TLR4, thereby indirectly inhibiting LPS-induced reactions downstream (Figure 1) [82,93].

## 6. Challenges and Strategies for Antibacterial and Antiendotoxic Marine Peptides or Proteins

Antibacterial/antiendotoxic peptides or proteins have several limitations, including toxicity, stability and cost in application to clinical cases. The following different strategies, including fusion expression, residue substitution, reducing hydrophobicity, cyclization or amidation and cost-effective purification method, have been developed to overcome these limitations.

### 6.1. Toxicity

Mason et al. studied the role of the C-terminal sequence of Chrysophsin-1 from the red sea bream and found that the toxicity of truncated Chrysophsin-1 in human lung fibroblast MRC-5 cells were reduced by 2.9 times after the removal of the RRRH sequence at its C-terminus [21]. D-amino acid substitution at the Leu9 position in Pro (3) TL, the frog skin peptide temporin L (TL, 13-residues long) analog, reduced the hemolytic activity and preserved the strong anti-Candida activity (Table 4) [94]. It has also been reported that the shortening of the peptide chain length of arenicin-1 (17-residues) is an effective assay to diminish its hemolytic activity [33]. Tripathi et al. found that Pro-substituted Chrysohphsin-1 analogs with unchanged physiochemical properties displayed significantly decreased cytotoxicity against human red blood cells and mammalian NIH-3T3 cells [20]. The first disulfide bond in the arenicin-3 derivative-NZ17074 was deleted, fused with a SUMO partner and expressed in *P. pastoris*. The result showed that the production level of N6 was increased by 1.4-fold compared to NZ17074, indicating the reduced toxicity to host cells after removing the first disulfide bond and fusing with SUMO [37]. Hydrophobicity of LPS-neutralizing antibacterial peptides, such as arenicin-1 or proteins, is closely related to cytotoxicity. Decreases in hydrophobicity can reduce the toxicity of peptides or proteins [32,34]. Additionally, topical application of marine peptides is preferred as a strategy to overcome host toxicity, immunogenicity and instability when exposed to host proteases (Table 4) [95].

### 6.2. Stability

C-terminal amidation of ShK from the sea anemone enhanced resistance to exoproteases (Table 4) [96]. N-terminal acylation of msHemerycin, which was isolated from the lugworm *Marphysa sanguinea*, showed stronger antibacterial activity due to increasing lengths of fatty acids and improved stability from proteolytic degradation by some peptidases [97]. In our study, the C-terminal amidation of N6 enhanced the antibacterial activity against *S. typhimurium* in mice and the stability by 3-fold at low pH (data not published), which may be associated with increasing positive charges in the peptide and with stabilizing the amphipathic helix formation [99]. Additionally, cyclization is commonly used as a strategy in the pharmaceutical industry to constrain the conformation of AMPs, such as lactoferricin, which can increase the antibacterial activity [98]. Likewise, backbone cyclization of the conotoxin MII from marine snails of the *Conus* genus by using a range of linkers that greatly improved the resistance to proteases, such as EndoGluC [100], which may be due to a more constrained structure that is less susceptible to protease degradation [98]. An all D-amino acid analog of 25-residue pleurocidin, which was derived from the winter flounder *Pleuronectes americanus*, showed improved activity against fungi and breast cancer cells, increased proteolytic resistance against proteases, such as trypsin, plasmin and carboxypeptidase B, and dramatically decreased the hemolytic activity [101,102,103].

### 6.3. Cost

To provide large quantities of peptides for clinical trials, it is necessary to find efficient production methods of peptides. Short and simple structural peptides with sufficient stability and biological activity can be efficiently produced by chemical synthesis but may be limited by expensive costs. Comparably, heterologous expression may be a more effective and practical way to obtain the bioactive peptides at a large scale. Particularly, the fungal fusion expression system is very favorable and helpful to obtain a high yield of target peptides by enhancing the solubility of peptides and the efficiency in production [104]. As seen in the examples of NZ17074 and N6, SUMO is successfully applied to increase the solubility and yield of peptides [37,67]. Additionally, improvement in solvent extraction techniques (such as supercritical fluid extraction, pressurized solvent extraction and microware/ultrasound/pulsed electric field/enzyme-assisted extraction) and the cost–effective purification methods including using monolithic columns and the intein system in AMP production can also be introduced to reduce the production cost for antibacterial/antiendotoxic peptides or proteins (Table 4) [17,105,106,107]. 

Of note, chemical synthesis, although relatively complex and costly, enables the incorporation of non-natural functionality, such as D-amino acids and acylation modification, into peptides to improve their activity [38,39,66]. With the development of new technologies, chemical synthesis may receive more and more attention in peptide production in the future. 

## 7. Conclusions

A large number of bioactive natural products, including peptides and proteins, have been found from the large marine natural resource library. The slower pace of antibacterial/antiendotoxic peptides or proteins in clinical trials may be due to stability, high production cost, and unknown toxicity. However, topical application of marine peptides or proteins may be the most promising development in clinical practice, with a deeper understanding of the mechanisms of action and cost-effective production progress in the near future. 

## Figures and Tables

**Table 1:** Antibacterial and antiendotoxic peptides or proteins from marine organisms and their characteristics.

Peptides/Proteins	Residues	Charge (+)	PI	Structures	GRAVY	References
Hc-CATH	30	12	12.61	α-helix	−0.273	[18]
Chrysophsin-1, 2, and 3	25, 25, 20	5, 5, 4	NN	α-helix	NN	[19]
Chrysophsin-1 variants	25	5	NN	α-helix	NN	[20]
ALP1, ALP2	17	6, 4	11.35, 9.3 ^a^	β-sheet	−0.729, 0.094 ^a^	[33]
Arenicins-1, 2, and 3	21	6, 6, 4	10.83, 10.85, 9.25 ^a^	β-sheet, β-turn	−0.07, −0.057, −0.048 ^a^	[26,35]
NZ17074	21	4	9.37	β-sheet	−0.243	[36]
N2	21	4	9.38	α-helix, β-sheet	−0.033	[36]
N6	21	4	10.72	α-helix, β-sheet	−0.310	[36]
CNC, N6NH2	32, 21	12, 5	NN, 11.64	NN, β-sheet	NN	No publication ^b^
Ogipeptins A, B, C, and D	NN	NN	NN	Cyclic peptides	NN	[38]
Pa4	33	1	8.59	α-helix	0.745	[49]
LALF	101	9	10.09	α-helix, β-sheet	−0.552	[40]
Piscidins-1, -2, and -3	22	3.4	12.01 ^a^	Random structure	−0.59	[50]
Hydrostatin-TL1 and -SN1	9	NN	NN	α-helix	NN	[22,23,24]

NN: no data; ^a^ PI and GRAVY were calculated by http://web.expasy.org/protparam/ and http://www.gravy-calculator.de/; ^b^ data not published in our work.

**Table 2:** Antimicrobial activity of antibacterial and antiendotoxic peptides or proteins from marine organisms.

Peptides/Proteins	Natural Products/Derivatives	Sources	Antimicrobial Spectrum	MIC (μM)	Status	References
Chrysophsins-1, -2 and -3	Natural products	Red sea bream: *Chrysophrys major*	G^−^: *E. coli*, *Vibrio*, *Aeromonas salmonicida* G^+^: *Bacillus subtilis*, *Lactococcus garvieae*, *Streptococcus iniae*	1.25~10 1.5~10	NN	[19]
Chrysophsin-1 variants	Derivatives	Chemical synthesis	G^−^: *E. coli*, *P. aeruginosa*, *Klebsiella pneumonia*; G^+^: *B. subtilis*, *S. aureus*, MRSA Fungi: *Candida*, *Cryptococcus neoformans*, *Sporothrix schenckii*	3.0~27.2 0.8~6 3~27.2	NN	[20]
Hydrostatins-TL1 and -SN1	Natural products	Sea snake: *Hydrophis cyanocinctus*; Chemical synthesis	NN	NN	Preclinical: colitis induced by LPS or dextran sodium sulfate; acute lung injury (ALI) against LPS	[22,23,24]
Arenicins-1, -2, and -3	Natural product	Marine lugworm: *Arenicola marina*	G^−^: *E. coli*, *P. aeruginosa*, *S. typhimurium* G^+^: *S. aureus*, *S. epidermidis*, *E. faecium* Fungi: *C. albicans*, *C. parapsilosis*, *Malassezia furfur*, *Trichosporon beigelii*, *T. rubrum*	2~8 2~8 4.5~9	Preclinical: UTI against *E. coli*; septicemia against *E. coli* and *P. aeruginosa*; thigh infections against *E. coli* Company: Adenium Biotech Copenhagen	[25,26,27,29,30,31,33]
AA139	Arenicin-3 derivative		G^−^: *E. coli*, *P. aeruginosa*, *Klebsiella pneumoniae*, *Acinetobacter baumannii*		FIM clinical I	[51]
Arenicin-1 variants	Arenicin-1 derivatives	Recombinant expression	G^−^: *E. coli*, *P. aeruginosa* G^+^: *S. aureus*	0.8~50 3.13~50	NN	[32]
ALP1, ALP2	Arenicin-1 derivatives	Recombinant expression	G^−^: *E. coli*, *P. aeruginosa*, *K. pneumoniae* G^+^: MRSA	0.5~4 0.06~0.12	NN	[33]
Arenicin-1	Natural product	Recombinant expression	G^−^: *E. coli*, *P. aeruginosa*, *K. pneumoniae* G^+^: *B. subtilis*, *S. aureus*	0.16~1.25 0.31~0.62	NN	[34]
Ar-1[V8R]	Arenicin-1 derivative	Recombinant expression	G^−^: *E. coli*, *P. aeruginosa*, *K. pneumoniae* G^+^: *B. subtilis*, *S. aureus*	0.08~1.25 0.62	NN	[34]
NZ17074	Arenicin-3 derivative	Chemical synthesis	G^−^: *E. coli*, *S. typhimurium*, *S. pullorum*, *S. choleraesuis*, *P. aeruginosa* G^+^: *S. aureus*, *S. suis*, *B. subtilis* Fungi: *C. albicans*	0.01~0.16 0.01~0.65 0.65	Preclinical: UTI against *E. coli*; thigh infections against *E. coli*; peritonitis/sepsis against *E. coli*	[35,37]
N2, N6	NZ17074 derivatives	Chemical synthesis	G^−^: *E. coli*, *S. typhimurium*, *S. pullorum*, *S. choleraesuis*, *P. aeruginosa* G^+^: *S. aureus*, *S. suis*, *B. subtilis* Fungi: *C. albicans*	0.01~0.16 0.01~0.65 1.3~2.6	Preclinical: peritonitis against *E. coli*; peritonitis against *S. enteritidis*; endotoxemia against LPS	[36,37]
CNC, N6NH2	Modification of N6	Chemical synthesis	G^−^: *S. typhimurium*	0.81~1.7	Preclinical: peritonitis against *S. enteritidis*	No publication ^f^
Ogipeptin A, B, C and D	Natural products	Marine bacterium: *Pseudoalteromonas*	G^−^: *E. coli* G^+^: *S. aureus*	0.25~1 ^a^ 8~128 ^a^	NN	[38,39]
ALFP*m*3	Natural product	The black tiger shrimp: *Penaeus monodon*	G^−^: *Vibrio*, *Salmonella*, *E. coli*, *Enterobacter cloacae*, *Erwinia carotovora* G^+^: *S. aureus*, *Bacillus*, *Micrococcus*, *Aerococcus* Fungi: *Fusarium oxysporum*, *B. cinerea*, *P. crustosum* Virus: WSSV	0.095~50 0.19~100 1.56~25	NN	[43,66]
SpALF6	Natural product	Mud crab: *Scylla paramamosain*	G^−^: *Vibrio*, *Aeromonas*, *E. coli* G^+^: *S. aureus*, *Bacillus* Fungi: *Pichia*, *Candida*	<6.25 6.25~12.5 12.5~25	NN	[47]
Hc-CATH	Natural product	Sea snake: *Hydrophis cyanocinctus*	G^−^: *E. coli*, *Klebsiella*, *Shigella*, *Pseudomonas*, *Salmonella*, *Proteus*, *Vibrio*, *Edwarsiella*, *Aeromonas* G^+^: *S. aureus*, *Bacillus*, *Enterococcus*, *Nocardia* Fungi: *Candida*, *Arcyria*	0.16~10.33 1.29~20.67 1.29~2.59		[18]
Pardaxin	Natural product	Moses sole fish: *Pardachirus marmoratus* and *P. pavoninus*	G^−^: *E. coli*, *S. typhimurium*, *A. calcoaceticus*, *P. aeruginosa* G^+^: *S. aureus*, *S. epidermidis*, *B. megaterium*, *M. luteus*, *B. subtilis*	1~40 1~10	NN	[48,50]
Piscidins-1, -2, and -3	Natural product	Fish: hybrid striped bass	G^−^: *E. coli*, *P. aeruginosa*, *S. typhimurium*, *K. pneumonia*, *Aeromonas*, *Shigella* G^+^: *S. aureus*, *S. epidermidis*, *B. subtilis, Lactococcu*, *Streptococcus* Fungus: *C. albicans*, *M. furfur*, *T. beigelii* Parasites: ciliates, dinoflagellate Cancer: HeLa, HT1080 cells	0.8~25 1~12.5 1.56~12.5	Preclinical: peritonitis against LPS	[50,52,53,54,55,56,57]
Piscidin-1 analogues	Piscidin-1 derivatives	Chemical synthesis	G^−^: *E. coli*, *S. typhimurium*, *P. aeruginosa*, *K. pneumoniae* G^+^: *S. aureus*, *S. epidermidis*, *B. subtilis*	1~24 1~16	Preclinical: peritonitis against LPS	[50,56,57]
Phosvitin (Pv)	Natural product	Fish: *D. rerio*	G^−^: *E. coli*, *A. hydrophila* G^+^: *S. aureus*	3~3.1 ^b^ 3 ^b^	Preclinical: sepsis against LPS	[58,65]
Pt5	Pv derivative	Recombinant expression	G^−^: *A. hydrophila*	NN	Preclinical: zebrafish against *A. hydrophila* ^c^	[59]
Pt5e	Pt5 derivative	Recombinant expression	G^−^: *E. coli* G^+^: *S. aureus*	1.2 1.8	Preclinical: sepsis against LPS	[60]
ZRANB2	Natural product	Zebrafish *D. rerio*; recombinant expression	G^−^: *E. coli*, *V. anguillarum*, *A. hydrophila*	9.7 ^d^	Preclinical: embryos challenged with *A. hydrophila* ^e^	[61]
Z_1/37_, Z_11/37_, Z_38/198_	ZRANB2 derivatives	Chemical synthesis; recombinant expression	G^−^: *E. coli*, *V. anguillarum*, *A. hydrophila*	8.5~9.3 ^d^	Preclinical: embryos challenged with *A. hydrophila* ^e^	[61]
Ls-Stylicin1	Natural product	The Pacific blue shrimp: *L. stylirostris*; recombinant expression	G^−^: *Vibrio splendidus* LGP, *Vibrio penaecidae*, *Vibrio nigripulchritudo* Fungi: *F. oxysporum*	40~80 1.25~2.5	NN	[62]

NN: no data; ^a^ MIC: μg/mL; trinary tract infection (UTI); ^b^ IC_50_: μM; ^c^ zebrafish model; ^d^ IC_50_: μg/mL; ^e^ embryos challenged with *Aeromonas* *hydrophila*; ^f^ data not published in our work.

**Table 3:** Heterologous expression of antibacterial and antiendotoxic marine peptides and proteins in microorganisms.

Peptides/Proteins	Expression	Carrier proteins	Vectors	Yields (mg/L)	Purity (%)	References
Arenicin-2	*E. coli*	KSI, CBD, and TrxA	pET-32a(+)	5	NN	[63]
Arenicin-1 variants	*E. coli*	Modified TrxA (M37L)	pDNA	1~4	NN	[32]
ALP1, ALP2	*E. coli*	TrxL	pBR322	7.5~9	NN	[33]
Arenicin-1	*E. coli*	Modified TrxA (M37L)	pDNA	4.2	NN	[34]
Ar-1[V8R]	*E. coli*	Modified TrxA (M37L)	pDNA	8.5	NN	[34]
Piscidin 1, piscidin 3	*E. coli*	TrpLE	TrpLE	1	>90	[64]
Pv	*E. coli*	Thioredoxin	pET28a	NN	NN	[65]
Pt5	*E. coli*	Thioredoxin	pET28a	NN	NN	[59]
Pt5e	*E. coli*	Thioredoxin	pET28a	NN	NN	[60]
ZRANB2, Z_38_/_198_	*E. coli*	NN	pET28a	NN	NN	[61]
Ls-Stylicin1	*E. coli*	His6	pET-28b(+)	NN	NN	[62]
SpALF6	*E. coli*	His6	pET30a	NN	NN	[47]
ALF*m*3	*P. pastoris*	NN	pPIC9K	118.4	NN	[43]
NZ17074	*P. pastoris*	SUMO3	pPICZaA	4.1	90	[67]
N6	*P. pastoris*	SUMO3	pPICZaA	9.7	NN	[37]

KSI: ketosteroid isomerase; CBD: cellulose-binding domain; TrxA: thioredoxin A; NN: no data.

**Table 4:** Challenges and strategies for new antibacterial and antiendotoxic marine peptides or proteins.

Challenges	Strategies	References
Toxicity	Amino acids substitution (including D-amino acids) or deletion; truncation	[20,21,32,37,94]
	Fusion expression	[37]
	Reducing hydrophobicity	[32,34]
	Topical application	[95]
Stability	Amidation, acetylation and cyclization	[96,97,99]
	D-amino acids substitution	[101,102,103]
Cost	New fusion expression system	[37,67]
	Improvement in solvent extraction technique	[17,105]
	Cost-effective purification method	[106,107]
